# Femoral nerve compression by an iliopsoas bursal cyst coexisting With lumbar spinal stenosis: a case report

**DOI:** 10.3389/fsurg.2026.1948532

**Published:** 2026-10-23

**Authors:** Yongdong Zhou, Shanzhi Li, Hongyi Kang, Bangjian Zhou, Wenping Liu, Haoduan Dou, Rui Tang

**Affiliations:** 1 Shapingba District Hospital of Traditional Chinese Medicine, Chongqing, China; 2 Clinical Medical College, Chengdu University of Traditional Chinese Medicine, Chengdu, Sichuan, China

**Keywords:** case report, femoral nerve compression, iliopsoas bursal cyst, lumbar spinal stenosis, open cyst excision

## Abstract

**Background:**

Iliopsoas bursal cysts are rare synovial proliferative lesions, most commonly associated with osteonecrosis of the femoral head, osteoarthritis, and rheumatoid arthritis. When a cyst compresses the femoral nerve, it may cause groin pain, radiating anterior thigh pain, and an antalgic gait, closely mimicking lumbar spinal stenosis (LSS). Only approximately 20 comparable cases of femoral nerve compression have been documented worldwide. Concomitant LSS and femoral nerve compression by an iliopsoas bursal cyst have not previously been reported. The coexistence of LSS and femoral nerve compression caused by an iliopsoas bursal cyst may result in delayed or missed diagnosis.

**Case presentation:**

We report a patient with concomitant LSS and femoral nerve compression caused by an iliopsoas bursal cyst who was initially treated for LSS alone. After 3 days of integrative conservative treatment for LSS, the low-back, posterior thigh, and lateral calf pain improved substantially; however, the right anterior thigh and groin pain became intolerable. Subsequent hip ultrasonography and magnetic resonance imaging (MRI) demonstrated an iliopsoas bursal cyst adjacent to the femoral nerve. Available electromyography findings showed a reduced right femoral nerve response amplitude. Considering the patient's overall clinical presentation, we considered right femoral nerve injury to be the most likely diagnosis. The patient underwent open anterior cyst excision to achieve complete neural decompression. The anterior thigh pain improved rapidly, with the visual analog scale (VAS) score decreasing from 8 to 2 on postoperative day 1. No symptom recurrence was observed during 12 months of follow-up.

**Conclusion:**

This case provides an important clinical warning. In older patients with chronic occupational musculoskeletal overuse, extraspinal femoral nerve compression should be considered when treatment for LSS relieves low-back and posterior thigh pain but severe anterior thigh or groin pain persists or worsens. Hip Doppler ultrasonography, MRI, and electromyography/nerve conduction studies should be performed to exclude extraspinal femoral nerve compression. In patients with severe cyst-related femoral nerve compression, open cyst excision can provide rapid neural decompression.

## Introduction

The iliopsoas bursa is the largest synovial bursa in the human body. It is present in approximately 98% of adults, measures an average of 6 × 3 cm, and lies between the iliopsoas tendon and the hip joint at the medial aspect of the femoral triangle. It reduces friction during iliopsoas movement and facilitates tendon excursion (1, 2). Under normal conditions, the bursa is collapsed and contains only a small amount of synovial fluid, making it difficult to visualize on imaging. Excessive friction or compression may induce acute inflammation of the bursal wall, resulting in synovial edema, hyperemia, thickening or fibrosis, increased fluid production, and cyst formation (3). Although the iliopsoas bursa is anatomically adjacent to the femoral nerve, femoral nerve compression by an iliopsoas bursal cyst is rare. Only approximately 20 similar cases have been reported, and the symptoms are usually nonspecific (4). To our knowledge, concomitant LSS and femoral nerve compression by an iliopsoas bursal cyst has not previously been reported. Reported manifestations include lower-extremity weakness, groin tenderness, radiating neuropathic pain, and restricted mobility. These findings overlap with those of LSS, lumbar disc herniation, and hip synovitis, increasing the risk of delayed or missed diagnosis when the disorders coexist (5–7). Here, we describe a patient with LSS and an iliopsoas bursal cyst compressing the femoral nerve. Treatment directed at LSS relieved the low-back, posterior thigh, and lateral calf pain, but the right anterior thigh and groin pain became intolerable. Subsequent evaluation identified femoral nerve compression by the cyst, which was successfully treated by surgical excision. The participant provided written informed consent to participate. Written informed consent was also obtained for publication of potentially identifiable images and clinical data.

## Case presentation

A 75-year-old male farmer was admitted with a 1-week history of low-back pain radiating to the right lower extremity and restricted mobility. The pain had developed without an obvious precipitating event and radiated to the posterior right thigh and lateral right calf; only mild anterior right thigh pain was initially present. The pain was distending in character, improved with rest, and worsened with activity. Walking was limited: after approximately 100 steps, he had to stop because of right-leg pain before continuing, consistent with intermittent claudication, although he could stand without difficulty. He had no relevant medical history. His occupation involved many years of strenuous physical work with repetitive bending.

On admission, no obvious scoliosis was observed. Marked tenderness between the L4 and L5 spinous processes reproduced pain radiating to the posterior right thigh and lateral calf, and the right buttock was tender. The straight-leg-raising and Bragard tests were positive on the right. The femoral nerve stretch test was performed with the patient prone and the lower extremity in a neutral position; the examiner stabilized the pelvis, flexed the knee to 90 degrees, and extended the hip while monitoring for radiating anterior thigh pain. The test produced only mild discomfort and no definite positive response. Muscle tone, strength, and sensation in both lower extremities were normal. Physiological reflexes were intact, and no pathological reflexes were elicited. The VAS score for low-back, posterior right thigh, and lateral calf pain was 7. Lumbar MRI showed disc bulging at L3–L4, L4–L5, and L5–S1, with ligamentum flavum hypertrophy and canal narrowing at L3–L4 and L4–L5. Although the initial radiology report did not use a formal quantitative grading system or document severe right L2–L4 nerve root compression, LSS was diagnosed initially based on the imaging findings. We attributed the low back pain and radiating right leg pain to lumbar disease; the mild anterior thigh pain did not initially receive adequate attention.

Initial conservative management combined Western medication with external traditional Chinese medicine therapies. Intravenous lappaconitine hydrobromide and parecoxib sodium were administered for analgesia, intravenous methylprednisolone for inflammation, and oral pethidine and other analgesics for pain control. Adjunctive physical therapies included medium-frequency electrotherapy and herbal compresses, intended to improve local circulation and relieve pain. After 3 days, pain in the low back, posterior right thigh, buttock, and lateral calf had markedly decreased, with a VAS score of 3. In contrast, the anterior right thigh pain persisted and became intolerable, severely limiting standing and daily activities. Even while holding the bed rail, the patient could stand for less than 3 min and walk fewer than five steps, with severe groin pain while walking. He remained in bed in a forced posture with the right hip and knee flexed and the right lower extremity adducted. The VAS score for anterior thigh pain was 8.

Repeat examination showed that L4-L5 interspinous tenderness with posterior thigh radiation, right buttock tenderness, and lateral calf tenderness were less pronounced than at admission. However, marked right-groin tenderness radiating to the anterior thigh was present. The straight-leg-raising and Bragard tests were now negative, whereas the femoral nerve stretch test and Patrick (FABER) test were positive. Cutaneous sensation over the anterior right thigh was reduced compared with the left, and the right patellar reflex was diminished. Iliopsoas strength was Medical Research Council grade 5/5; quadriceps strength could not be reliably assessed because of pain. A coexisting right-hip disorder was suspected, and MRI and Doppler ultrasonography of the right hip were obtained.

Hip MRI demonstrated fluid distension of the right iliopsoas bursa, cystic nodules, and right iliopsoas bursitis. Doppler ultrasonography identified two anechoic areas beneath the tendon sheath adjacent to the right femoral head, measuring approximately 31.2 × 7.5 mm and 11.7 × 2.3 mm. The larger lesion was irregular but well circumscribed, showed good acoustic transmission and internal septations, and contained no detectable blood-flow signal on color Doppler flow imaging. In conjunction with the clinical symptoms and examination findings, we considered that compression of the femoral nerve by a right iliopsoas bursal cyst was highly likely. Reduced cutaneous sensation over the anterior right thigh and a diminished right patellar reflex indicated progressive neurological impairment. Subsequent electromyography testing showed reduced right femoral nerve response amplitude. Although a complete evaluation—including bilateral femoral nerve compound muscle action potential amplitudes and distal latencies, saphenous nerve sensory conduction studies, and needle electromyography of the quadriceps, iliopsoas, and lumbar paraspinal muscles—was not performed, the combination of severe right groin pain during walking, marked right groin tenderness on examination, and magnetic resonance imaging and Doppler ultrasonographic findings led us to consider right femoral nerve injury; intraoperative and postoperative findings further supported this diagnosis. The right-hip MRI and Doppler ultrasonographic findings are shown in Figure 1.

**Figure 1:**
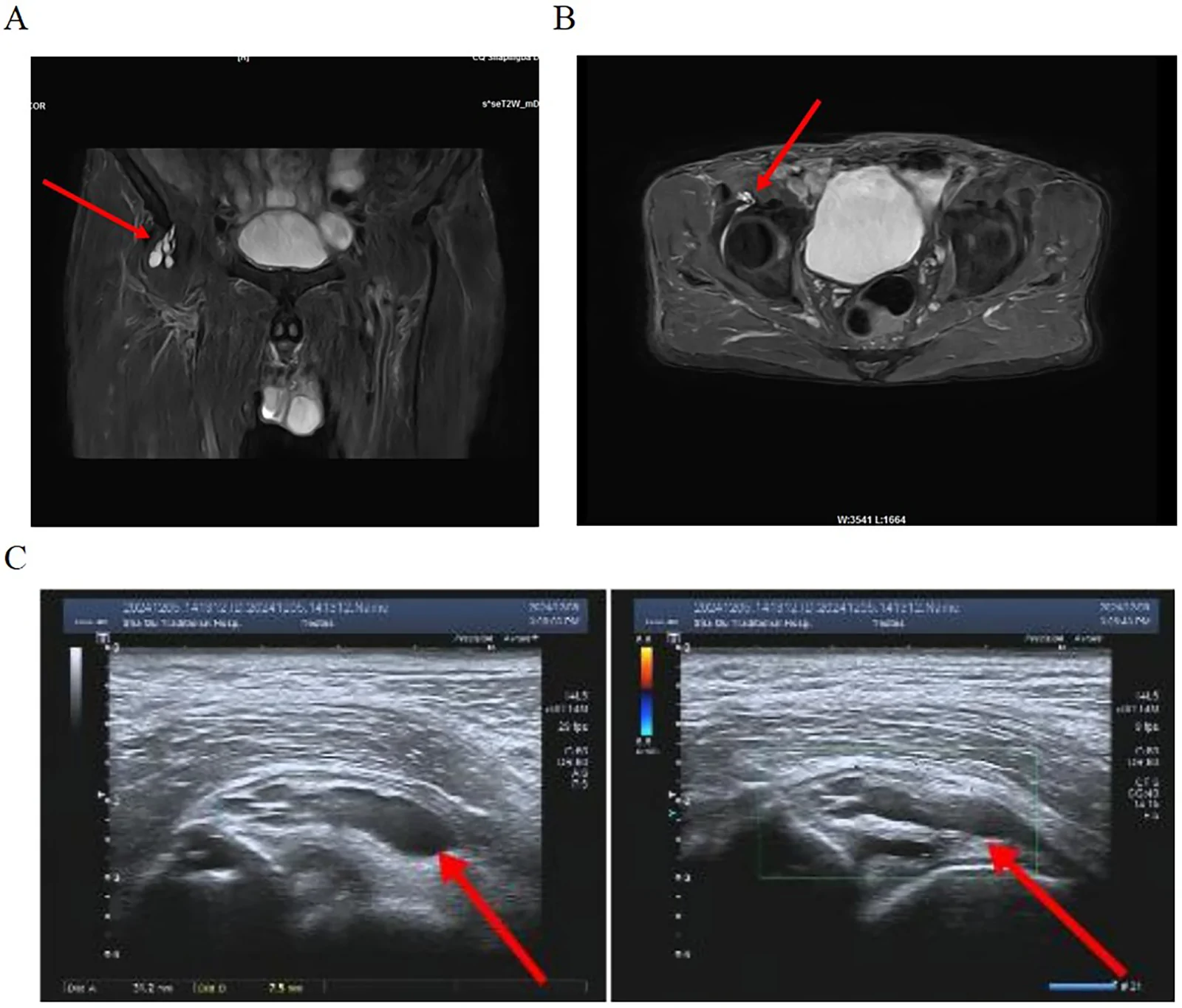
Preoperative magnetic resonance imaging (MRI) and Doppler ultrasonography of the right hip. **(A)** Coronal T2-weighted MRI of the pelvis showing a hyperintense cystic lesion anterior to the right hip joint. **(B)** Axial T2-weighted MRI of the right hip showing the relationship between the cyst and the surrounding structures; the base of the cyst communicates with the iliopsoas Bursa. **(C)** Grayscale ultrasonography and color Doppler ultrasonography showing a well-defined anechoic lesion closely adjacent to the femoral nerve. Red arrows indicate the iliopsoas bursal cyst, and the femoral nerve courses nearby.

Management options, including continued conservative treatment, ultrasound-guided aspiration with corticosteroid injection, and surgery, were discussed with the patient. Because he was a farmer with limited financial resources and requested immediate definitive treatment, treatment selection was individualized accordingly. Given the relatively large cysts and the patient's preference for rapid definitive treatment, ultrasound-guided aspiration was not selected; hip arthroscopy was also excluded because of cost. After written informed consent was obtained, open anterior cyst excision was performed. Intraoperatively, two relatively large, rounded cystic masses, each approximately 2.0 cm in diameter, were found compressing the adjacent muscles and nerve, and the iliopsoas muscle was markedly tense. After adequate exposure, the bases of the cysts were found to communicate with the iliopsoas bursa. Following complete excision, the femoral nerve appeared hyperemic and edematous. The communicating bases were ligated and closed. Histopathological examination confirmed iliopsoas bursal cysts. The intraoperative findings and surgical steps are shown in Figure 2.

**Figure 2:**
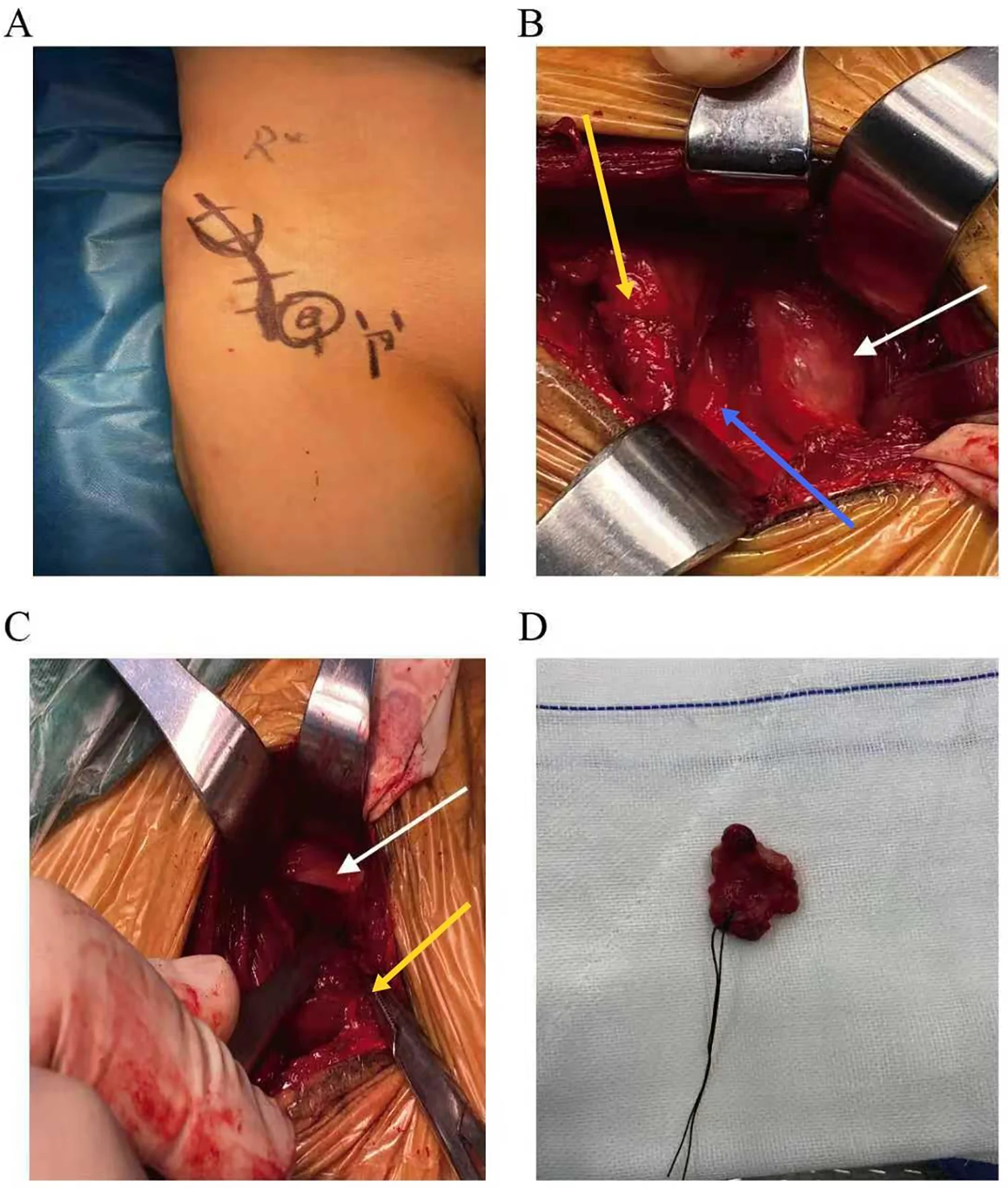
Intraoperative findings and surgical specimen. **(A)** Preoperative skin marking indicating the planned incision site. **(B)** Intraoperative view showing exposure of the iliopsoas bursal cyst (white arrow), tensor fasciae latae (yellow arrow), and rectus femoris (blue arrow). **(C)** After complete cyst excision, the decompressed femoral nerve is visible (white arrow); the yellow arrow indicates the iliopsoas muscle, which is retracted with a hemostatic forceps. **(D)** Gross appearance of the resected cystic mass submitted for histopathological examination.

On postoperative day 1, the patient reported marked relief of the right anterior thigh pain and only mild incisional pain. The forced posture had resolved. The VAS score was 2 for anterior thigh pain and 1 for low-back, posterior thigh, and lateral calf pain. By postoperative day 2, he could stand and walk unaided for 30 min without significant discomfort; the VAS score for anterior thigh pain was 1. The straight-leg-raising, Bragard, and femoral nerve stretch tests were all negative. At the 1-, 3-, and 6-month follow-up visits, the initial symptoms and associated clinical findings had completely resolved, with a VAS score of 0. At 3 months postoperatively, the patient resumed light farm work. No recurrence of similar symptoms was observed during 1 year of follow-up. Unfortunately, for reasons including financial constraints, the patient declined repeat MRI and Doppler ultrasonography of the right hip.

## Discussion

The precise mechanism underlying iliopsoas bursal cyst formation remains incompletely understood. Available evidence links these lesions to osteoarthritis, osteonecrosis of the femoral head, and recurrent local inflammation; surgery, trauma, infection, and overuse have also been implicated (8, 9). Degenerative osteoarthritis and ischemic necrosis of the femoral head may increase intra-articular pressure and create an occult communication between the joint capsule and bursa, allowing synovial fluid to enter and enlarge the bursa. Systemic inflammatory disorders, including rheumatoid arthritis and gout, may also involve the iliopsoas bursa and promote cyst formation (10). We hypothesize that, in this older farmer, decades of repetitive bending may have repeatedly stretched and rubbed the hip muscles and bursa, leading to chronic local inflammation and possibly contributing to cyst formation.

Clinical manifestations depend on cyst size, location, and the degree of pressure exerted on adjacent structures. Small cysts may be asymptomatic and detected incidentally. As a cyst enlarges, a groin mass may become palpable. Femoral vein compression can cause lower-extremity edema and impaired venous return. Femoral nerve compression may cause radiating anterior thigh pain, sensory loss, and a diminished patellar reflex (11). Expansion during hip movement may also produce pain, restricted motion, and an antalgic gait (4). These manifestations overlap substantially with those of LSS, lumbar disc herniation, and other lumbar disorders. Differentiation is particularly difficult with upper lumbar lesions at L1-L2 or L2-L3 because the L2 and L3 nerve roots also supply the anterior thigh (12, 13). Examination should therefore include careful assessment for groin tenderness, restricted hip abduction, and a positive FABER test.

Doppler ultrasonography and MRI are central to imaging evaluation (14–16). Ultrasonography is convenient, noninvasive, and cost-effective; it delineates cyst location, size, morphology, and internal echogenicity, permits dynamic assessment during hip motion, and helps distinguish a cyst from other groin lesions such as hematoma or lymphadenopathy. MRI provides superior soft-tissue contrast and accurately defines the cyst margins and anatomical relationships with the hip joint and neurovascular structures. Iliopsoas bursal cysts are typically hyperintense on T2-weighted images, and MRI may demonstrate communication with the joint cavity (17). Combined ultrasonography and MRI can improve diagnostic confidence and reduce the risk of missing the lesion when only one modality is used (18, 19). When nerve compression is suspected, electromyography and nerve conduction studies can objectively document neural injury.

Small cysts may be managed with local physical therapy or CT- or ultrasound-guided aspiration followed by corticosteroid injection. These approaches are relatively simple but may have a higher recurrence rate and uncertain long-term efficacy (20). Surgical excision should be considered when the cyst compresses a nerve or vessel, causes substantial functional impairment, or produces progressively worsening symptoms (21). Options include open surgery and minimally invasive hip arthroscopy. Complete removal of the cyst and involved bursal tissue can fully relieve compression and may reduce recurrence. Treatment should be individualized according to lesion characteristics, available expertise, cost, and patient preference. In the present case, open anterior excision produced rapid symptom resolution, and no clinical recurrence was observed at 1 year, supporting the effectiveness of open decompression in a carefully selected patient.

This report has several limitations. First, although no recurrence occurred during 1 year of follow-up, longer-term outcomes are unavailable. Second, only open anterior excision was performed; direct comparison with image-guided aspiration and corticosteroid injection or arthroscopic surgery was not possible. Third, because the procedure was performed at a primary-level hospital lacking the necessary equipment and specialized personnel, neither complete preoperative electrodiagnostic testing nor intraoperative neurophysiological monitoring could be performed. In addition, histopathology confirmed a cyst but did not provide a detailed analysis of the cyst wall architecture or cellular composition.

## Conclusion

In patients presenting with low-back and leg symptoms, the differential diagnosis should not be restricted to lumbar disorders. Potential hip-region causes, including iliopsoas bursal cysts and piriformis syndrome, should also be considered. In patients with LSS or lumbar disc herniation who have persistent severe anterior thigh and groin pain together with a persistently positive femoral nerve stretch test, hip Doppler ultrasonography, MRI, and lower-extremity electrophysiological testing should be performed to avoid missed diagnosis. Depending on the individual circumstances, management may include conservative therapy, image-guided aspiration with corticosteroid injection, arthroscopic surgery, or open excision. The optimal strategy for patients with a bursal cyst coexisting with LSS remains uncertain and requires further investigation. Future studies should accumulate additional cases and systematically analyze risk factors for cyst formation and recurrence to strengthen the evidence base for diagnosis and treatment.

## Data Availability

The datasets presented in this study can be found in online repositories. The names of the repository/repositories and accession number(s) can be found in the article/Supplementary Material.
